# Supplementary material for: Excess p50 induces Arp1-dependent dynactin clusters containing the assembly factor VezA
Source: bioRxiv. 2026 Jun 2:2026.05.29.728734. Preprint. [Version 1] doi: 10.64898/2026.05.29.728734 (PMC13251980; doi:10.64898/2026.05.29.728734)

bioRxiv preprint doi: https://doi.org/10.64898/2026.05.29.728734; this version posted June 2, 2026. The copyright holder for this preprint (which was not certified by peer review) is the author/funder, who has granted bioRxiv a license to display the preprint in perpetuity. It is made available under a CC-BY 4.0 International license.

Table S1. *Aspergillus nidulans* strains used in this study

Strain	Genotype	Source
JZ788	Arp11-GFP-Af*pyrG, argB2::[argB*-alcAp*::mCherry-*RabA]; ΔnkuA::argB; pyrG89; pantoB100; yA2*	(Qiu et al., 2020)
RQ2	GFP-*nudA*^{HC}*; argB2::[argB*-alcAp*::mCherry-*RabA]; ΔnkuA::argB; pyrG89; pyroA*4; *yA2*	(Qiu et al., 2013)
RQ54	*argB2::[argB*-alcAp*::mCherry-*RabA]; ΔnkuA::argB; pyrG89; pyroA*4; *wA2*	(Qiu et al., 2013)
TNO2A3	*ΔnkuA::argB; pyrG89; pyroA*4	(Nayak et al., 2006)
XX222	GFP-*nudA*^{HC}*; argB2::[argB*-alcAp*::mCherry-*RabA]; ΔnkuA::argB; pantoB100*; *yA2*	(Zhang et al., 2014)
XY41	p25-GFP-AfpyrG; *ΔnkuA::argB; pyrG*89; *pyroA4*	(Zhang et al., 2011)
XY42	*argB2::[argB*-alcAp*::mCherry-*RabA]; ΔnkuA::argB; pyrG*89; *pantoB100*; *yA2*	(Qiu et al., 2018)
XY136	*ΔvezA-AfpyrG;* GFP-*nudA*^{HC}*; argB2::[argB*-alcAp*::mCherry-*RabA]; pyrG89; ΔnkuA::argB; pyroA*4; *yA2*	(Yao et al., 2015)
XY163	*vezA*-GFP-*AfpyrG; argB2::[argB*-alcAp*::mCherry-*RabA]; pyrG*89; *ΔnkuA::argB pyroA*4, *wA*2	(Yao et al., 2015)
XY167	ΔTM-*vezA*-GFP-*AfpyrG; argB2::[argB*- alcA(p)*::mCherry-*RabA; ΔnkuA::argB; pyrG89; pyroA4; wA2*	(Yao et al., 2015)
JZ706	p150-GFP-*AfpyrG; argB2::[argB*-alcAp*::mCherry-*RabA]; pyrG*89; *ΔnkuA::argB*	(Zhang et al., 2024)
JZ790	p62-GFP-Af*pyrG, argB2::[argB*-alcAp*::mCherry-*RabA]; ΔnkuA::argB; pyrG89; pantoB100; yA2*	(Zhang et al., 2024)
JZ942	Arp11-GFP-*AfpyrG; ΔvezA-AfpyrG; argB2::[argB*-alcAp*::mCherry-*rabA]; ΔnkuA::argB; pyrG89; yA2*	(Zhang et al., 2024)
JZ1025	*alcA*-Arp11; *argB2::[argB*- alcA(p)::mCherry-RabA]; ΔnkuA::argB, pyrG89; pantoB100; yA2*	(Zhang et al., 2024)
JZ1026	*alcA*-Arp1; *argB2::[argB*- alcA(p)::mCherry-RabA]; ΔnkuA::argB; pyrG89; pantoB100; yA2*	(Zhang et al., 2024)

bioRxiv preprint doi: https://doi.org/10.64898/2026.05.29.728734; this version posted June 2, 2026. The copyright holder for this preprint (which was not certified by peer review) is the author/funder, who has granted bioRxiv a license to display the preprint in perpetuity. It is made available under a CC-BY 4.0 International license.

JZ1034	*alcA*-p150; *argB2::[argB*- alcA(p)::mCherry-RabA], ΔnkuA::argB; pyrG89; pantoB100; yA2*	(Zhang et al., 2024)
JZ1122	Arp11-GFP-*AfpyrG*; p50-S-*AfpyrG, argB2::[argB*-alcAp::mCherry-rabA]; ΔnkuA::argB; pyrG89*	(Zhang et al., 2024)
JZ1123	Arp11-GFP-*AfpyrG*; p50-S-*AfpyrG; ΔvezA-AfpyrG*; *argB2::[argB*-alcAp::mCherry-rabA]; ΔnkuA::argB; pyrG89*	(Zhang et al., 2024)
JZ1125	p50-GFP-*AfpyrG*; *alcA*-Arp1; *argB2::[argB*-alcAp::mCherry-rabA]; ΔnkuA::argB; pyrG89, yA*	(Zhang et al., 2024)
SX1	p50-S-*AfpyrG; argB2::[argB*-alcAp::mCherry-rabA]; ΔnkuA::argB; pyrG89; pantoB100; yA2*	(Zhang et al., 2024)
SX2	p50-GFP-*AfpyrG; argB2::[argB*-alcAp::mCherry-rabA]; ΔnkuA::argB; pyrG89; pyroA4; wA2*	(Zhang et al., 2024)
XX370	p150-GFP-*AfpyrG; argB2::[argB*-alcAp*::mCherry-*RabA]*	(Zhang et al., 2024)
RQ413	*An2744*-mcherry-*AfpyrG*, *argB2::[argB*-alcAp*::mCherry-*RabA]; ΔnkuA::argB; pyrG*89; *pantoB100*; *yA2*	This work
JZ1134	*vezA*-GFP-*AfpyrG; alcA*-p50; ; *argB2::[argB*-alcAp*::mCherry-*RabA]; ΔnkuA::argB; pyrG*89; *yA2*	This work
JZ1137	*vezA*-GFP-*AfpyrG; alcA*-Arp11; *argB2::[argB*-alcAp*::mCherry-*RabA]; ΔnkuA::argB; pyrG*89; *yA2*	This work
JZ1138	*vezA*-GFP-*AfpyrG; alcA*-Arp1; *argB2::[argB*-alcAp*::mCherry-*RabA]; ΔnkuA::argB; pyrG*89; *yA2*	This work
JZ1155	*gpdA*-p50-S-*AfpyrG*; *argB2::[argB*-alcAp*::mCherry-*RabA]; ΔnkuA::argB; pyrG*89; *pantoB100*; *yA2*	This work
JZ1156	*vezA*-GFP-*AfpyrG; gpdA*-p50-S-*AfpyrG*; *argB2::[argB*-alcAp*::mCherry-*RabA]; ΔnkuA::argB; pyrG*89; *yA2*	This work
JZ1159	*gpdA*-p50-S-*AfpyrG*; *argB2::[argB*-alcAp*::mCherry-*RabA]; ΔnkuA::argB; pyrG*89; *pyroA4*; *wA2*	This work
JZ1160	p150-GFP-AfpyrG; *gpdA*-p50-S-*AfpyrG*; *argB2::[argB*-alcAp*::mCherry-*RabA]*	This work
JZ1168	*gpdA*-p50-GFP-*AfpyrG; argB2::[argB*-alcAp*::mCherry-*RabA]; ΔnkuA::argB; pyrG*89; *pantoB100*; *yA2*	This work
JZ1169	p50$^{\Delta 17\text{-}25}$-S-*AfpyrG; argB2::[argB*-alcAp*::mCherry-*RabA]; ΔnkuA::argB; pyrG*89; *pantoB100*; *yA2*	This work

bioRxiv preprint doi: https://doi.org/10.64898/2026.05.29.728734; this version posted June 2, 2026. The copyright holder for this preprint (which was not certified by peer review) is the author/funder, who has granted bioRxiv a license to display the preprint in perpetuity. It is made available under a CC-BY 4.0 International license.

JZ1171	p50$^{\Delta 17\text{-}25}$-S-*AfpyrG;* Arp11-GFP-*AfpyrG;* ; *argB*2*::[argB*-alcAp*::mCherry-*RabA]; ΔnkuA::argB; pyrG*89; *yA*2	This work
JZ1173	p50$^{\Delta 17\text{-}25}$-S-*AfpyrG;* p150-GFP-*AfpyrG;* ; *argB*2*::[argB*-alcAp*::mCherry-*RabA]; ΔnkuA::argB; pyrG*89; *yA*2	This work
JZ1175	p50$^{\Delta 17\text{-}25}$-S-*AfpyrG; vezA*-GFP-*AfpyrG;* ; *argB*2*::[argB*-alcAp*::mCherry-*RabA]; ΔnkuA::argB; pyrG*89; *wA*2	This work
JZ1184	p50-mCherry-*AfpyrG*; Δ*nkuA::argB; pyrG*89; *pyroA*4	This work
JZ1199	*gpdA*-p50-S-*AfpyrG*; Arp11-GFP-*AfpyrG*; Δ*vezA-AfpyrG; argB*2*::[argB*-alcAp*::mCherry-*RabA]*	This work
JZ1203	Arp11-mCherry; Δ*nkuA::argB; pyrG89; pyroA4*	This work
JZ1205	p50-mCherry; *gpdA-vezA*-GFP-*AfpyrG; yA2*	This work
JZ1222	Arp11-mCherry; *gpdA*-p50-GFP-*AfpyrG; yA2*	This work
JZ1223	Arp11-mCherry; *gpdA*-p50-S-*AfpyrG*	This work
JZ1227	Arp11-mCherry; *gpdA*-p50-S-*AfpyrG; pantoB*100	This work
JZ1229	Arp11-mCherry; *vezA*-GFP-*AfpyrG; gpdA*-p50-S-*AfpyrG, yA2*	This work
JZ1233	*alcA*-Arp1; *vezA*-GFP-*AfpyrG; gpdA*-p50-S-*AfpyrG;* Δ*nkuA::argB; pyrG*89; *yA*2	This work
JZ1236	*alcA*-Arp11; *vezA*-GFP-*AfpyrG; gpdA*-p50-S-*AfpyrG; argB*2*::[argB*-alcAp*::mCherry-*RabA]; ΔnkuA::argB; pyrG*89; *yA*2	This work
JZ1237	*alcA*-Arp1; Arp11-GFP-*AfpyrG; gpdA*-p50-S-*AfpyrG;* ; *argB*2*::[argB*-alcAp*::mCherry-*RabA]; ΔnkuA::argB; pyrG*89; *yA*2	This work
JZ1238	*alcA*-Arp1; Arp11-GFP-*AfpyrG;* ; *argB*2*::[argB*-alcAp*::mCherry-*RabA]; ΔnkuA::argB; pyrG*89; *yA*2	This work
JZ1240	*alcA*-p150; Arp11-GFP-*AfpyrG; pyrG*89; *yA*2	This work
JZ1251	*gpdA*-p50$^{\Delta 17\text{-}25}$-S-*AfpyrG; argB*2*::[argB*-alcAp*::mCherry-*RabA]; ΔnkuA::argB; pyrG*89; *pyroA4; wA*2	This work
JZ1255	*gpdA*-p50$^{\Delta 17\text{-}25}$-S-*AfpyrG;* Arp11-GFP-*AfpyrG;* ; *argB*2*::[argB*-alcAp*::mCherry-*RabA]; ΔnkuA::argB; pyrG*89; *yA*2	This work
JZ1257	*gpdA*-p50$^{\Delta 17\text{-}25}$-GFP-*AfpyrG; argB*2*::[argB*-alcAp*::mCherry-*RabA]; ΔnkuA::argB; pyrG*89; *pantoB*100*; yA*2	This work
XX331	*vezA*-GFP-*AfpyrG; pabaA*1; *wA*2	This work

bioRxiv preprint doi: https://doi.org/10.64898/2026.05.29.728734; this version posted June 2, 2026. The copyright holder for this preprint (which was not certified by peer review) is the author/funder, who has granted bioRxiv a license to display the preprint in perpetuity. It is made available under a CC-BY 4.0 International license.

XX1108	*gpdA*-p50-S-*AfpyrG*; p62-GFP-*AfpyrG*; *argB2::[argB*-alcAp*::mCherry-*RabA]; ΔnkuA::argB; pyrG*89; *yA2*	This work
XX1111	*gpdA*-p50-S-*AfpyrG*; Arp11-GFP-*AfpyrG*; *argB2::[argB*-alcAp*::mCherry-*RabA]; ΔnkuA::argB; pyrG*89; *yA2*	This work
XX1113	*gpdA*-p50-S-*AfpyrG*; GFP-*nudA*^{HC}; *argB2::[argB*-alcAp*::mCherry-*RabA]; ΔnkuA::argB; pyrG*89; *yA2*	This work
XX1115	*gpdA*-p50-S-*AfpyrG*; p25-GFP-*AfpyrG*; *argB2::[argB*-alcAp*::mCherry-*RabA]; ΔnkuA::argB; pyrG*89; *yA2*	This work
XX1119	*gpdA*-p50-S-*AfpyrG*; Arp11-GFP-*AfpyrG*; *argB2::[argB*-alcAp*::mCherry-*RabA]; ΔnkuA::argB; pyrG*89; *pyroA*4; *yA2*	This work
XX1123	*alcA-p50;* Arp11-GFP-*AfpyrG*	This work
XX1125	*gpdA*-p50-GFP-*AfpyrG*; *ΔvezA-AfpyrG; argB2::[argB*-alcAp*::mCherry-*RabA]; yA2*	This work
XX1129	*gpdA*-p50-GFP-*AfpyrG*; *alcA*-Arp1; *argB2::[argB*-alcAp*::mCherry-*RabA]; yA2*	This work
XX1132	*gpdA*-p50-GFP-*AfpyrG*; *alcA*-Arp11; *argB2::[argB*-alcAp*::mCherry-*RabA]; yA2*	This work
XX1133	*gpdA*-p50-GFP-*AfpyrG*; *alcA*-Arp11; *argB2::[argB*-alcAp*::mCherry-*RabA]*	This work
XX1135	*gpdA*-p50-S-*AfpyrG*; *vezA*$^{\Delta 1\text{-}20}$-GFP-*AfpyrG*; *argB2::[argB*-alcAp*::mCherry-*RabA]; wA2*	This work
XX1137	*gpdA*-p50-S-*AfpyrG*; *vezA*$^{\Delta 563\text{-}615}$-GFP-*AfpyrG*; *argB2::[argB*-alcAp*::mCherry-*RabA]; yA2*	This work
XX1139	*gpdA*-p50-S-*AfpyrG*; Arp11-GFP-*AfpyrG*; *ΔvezA-AfpyrG; argB2::[argB*-alcAp*::mCherry-*RabA]*	This work
XX1141	*gpdA*-p50-S-*AfpyrG*; ΔTM-*vezA*-GFP-*AfpyrG*; *argB2::[argB*-alcAp*::mCherry-*RabA]; ΔnkuA::argB; pyrG*89	This work
XX1142	p50-mCherry; *pabaA*1; *yA2*	This work
XX1145	*vezA*-GFP-*AfpyrG*; *gpdA*-p50-S-*AfpyrG*; *pantoB100*; *wA*2; yA2	This work
XX1146	*vezA*-GFP-*AfpyrG*; *gpdA*-p50-S-*AfpyrG*; *argB2::[argB*-alcAp*::mCherry-*RabA]; pantoB*100; *wA*2; yA2	This work
XX1148	Diploid of XX1142/XX1145	This work

bioRxiv preprint doi: https://doi.org/10.64898/2026.05.29.728734; this version posted June 2, 2026. The copyright holder for this preprint (which was not certified by peer review) is the author/funder, who has granted bioRxiv a license to display the preprint in perpetuity. It is made available under a CC-BY 4.0 International license.

XX1196	*vezA*-GFP-*AfpyrG*; *gpdA*-p50$^{\Delta 17\text{-}25}$-S-*AfpyrG; argB*2*::[argB**-*alcAp*::mCherry-*RabA]*	This work

bioRxiv preprint doi: https://doi.org/10.64898/2026.05.29.728734; this version posted June 2, 2026. The copyright holder for this preprint (which was not certified by peer review) is the author/funder, who has granted bioRxiv a license to display the preprint in perpetuity. It is made available under a CC-BY 4.0 International license.

Figure S1. Controls for the images from the strain containing Arp11-mCherry, VezA-GFP and *gpdA*-p50-S (see Figure 4E middle panels). The purpose of these controls is to show that the clustered VezA-GFP signals can be seen only in the green channel and the clustered Arp11-mCherry signals can be seen only in the red channel under our imaging conditions. Images of a strain containing VezA-GFP and *gpdA*-p50-S are shown in the left panels, and those of a strain containing Arp11-mCherry and *gpdA*-p50-S are shown in the right panels. Hyphal tip is indicated by a yellow arrowhead. Bar, 10 μm.

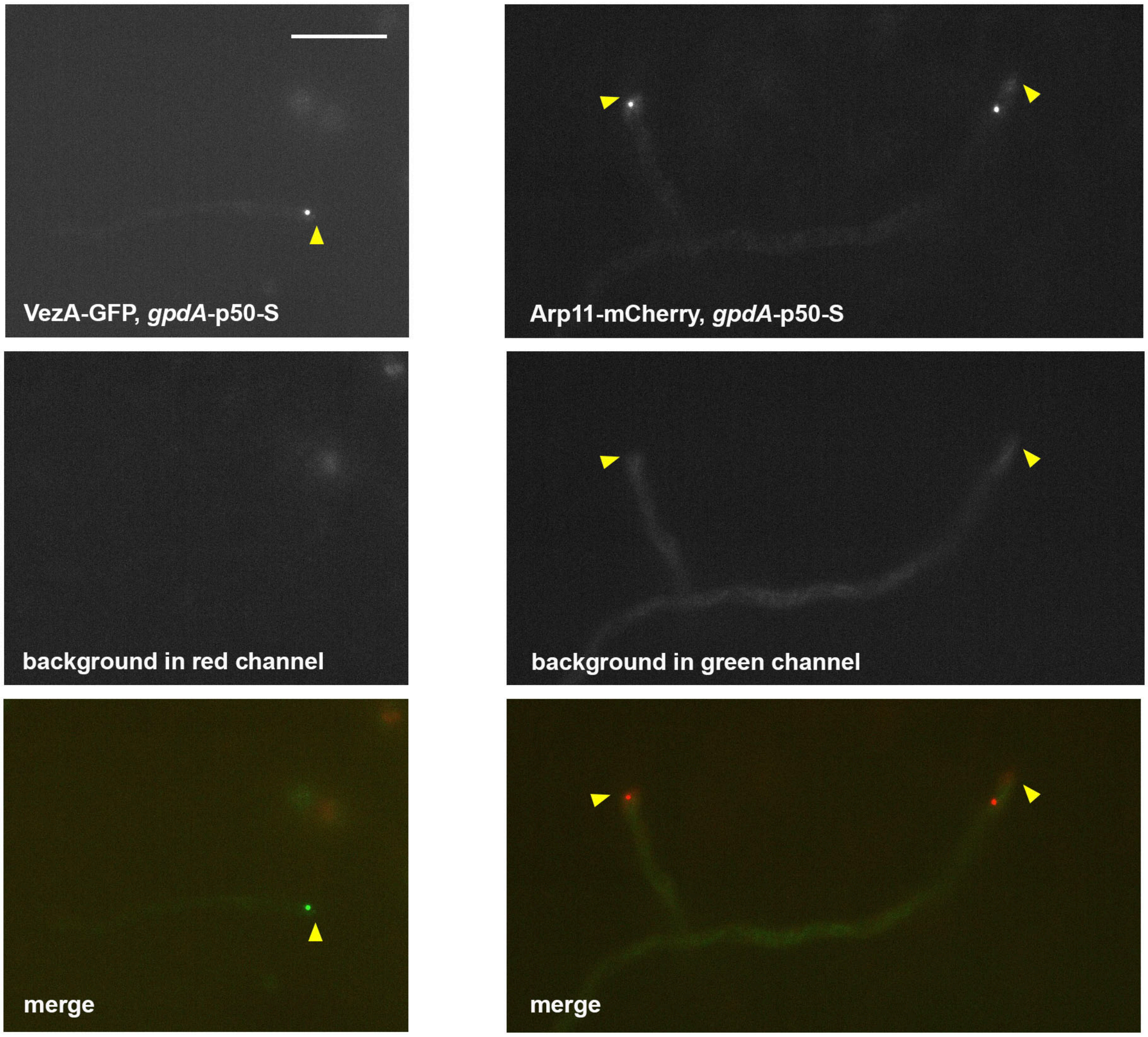

bioRxiv preprint doi: https://doi.org/10.64898/2026.05.29.728734; this version posted June 2, 2026. The copyright holder for this preprint (which was not certified by peer review) is the author/funder, who has granted bioRxiv a license to display the preprint in perpetuity. It is made available under a CC-BY 4.0 International license.

Figure S2. Arp11-GFP does not form clusters upon loss of p150. In the *alcA*-p150 background where the expression of p150 is repressed on glucose medium, Arp11-GFP signals are diffuse and slightly enriched at the hyphal tip but does not form clusters, which is in contrast to the cluster formation of Arp11-GFP in the *gpdA*-p50-S background (see Figure 2A and Figure 6A). Bar, 10 μm.

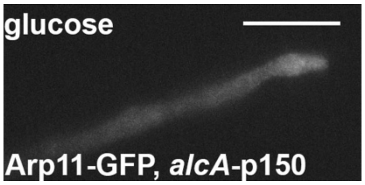

bioRxiv preprint doi: https://doi.org/10.64898/2026.05.29.728734; this version posted June 2, 2026. The copyright holder for this preprint (which was not certified by peer review) is the author/funder, who has granted bioRxiv a license to display the preprint in perpetuity. It is made available under a CC-BY 4.0 International license.

Figure S3. Protein sequence alignment of p50 proteins and beta-sheet structures of p50-Arp1 interaction. A sequence alignment of *A. nidulans* p50 and human p50. The amino acids involved in beta-sheet structures of p50-Arp1 interaction are highlighted by the cyan background. The p50 amino acids 17-25 (in both *A. nidulans* and human) around the first beta-sheet structure are highly conserved (yellow background). The alignment was done using the CLUSTAL O(1.2.4) multiple sequence alignment program. Residues that are identical (*), strongly similar (:) or weakly similar (.) are indicated. The structures of the porcine p50-Arp1 interactions shown below the alignment are based on published structure data from PBD 8PTK (Singh et al., 2024).

```
p50_A.nidu      MAFNKKYAGLPDLD-LAPDIYETPDLTDEASTVPTATLRTASNPDDTGSDSDIDREPVNA     59
p50_human       -MADPKYADLPGIARNEPDVYETSDLPEDDQAEFDAELEEL-------TSTSVEHIIVNP     52
                   : ***.**.:    **:*** ** :: .:   * *.         :.:.:::  **

p50_A.nidu      DEARAHFLGATVDARHVNFSDSIATKR-KSYRSKSRQRRKDTLADEEDSDEETESVERKL     118
p50_human       NAAYDKFKDKRVGTKGLDFSDRIGKTKRTGYESGEYEMLGEGLGVKE-------TPQQKY     105
                : *  :* .  *.:: ::*** *...: ..*.* . :   : *. :*       : ::*

p50_A.nidu      ARLQREVEELKDEMAASQNRPTSESGDVSGEAKDTSDDSVEKLSQALDNLYA-SSRGLLQ     177
p50_human       QRLLHEVQELTTEVEKIKTTVK-ES---ATEEKLTPVLLAKQLAALKQQLVASHLEKLLG     161
                 ** :**:**. *:   :.  . **   : * * *    .::*:   ::* *   . **

p50_A.nidu      PHSAAA-------LISRKLASAPTQASEVSGGQSKAAASTEAETTV--------PVAGIL     222
p50_human       PDAAINLTDPDGALAKRLLLQLE-ATKNSKGGSGGKTTGTPPDSSLVTYELHSRPEQDKF     220
                *.:*         * .* * .    :.: .**..  ::.*  ::::        *  . :

p50_A.nidu      SH---AASFDTRLALIESAMGISTSLNPFVTEGSSEPPLQPVLPALDHLNSRLSTLITLL     279
p50_human       SQAAKVAELEKRLTELETAVRCDQ---------DAQNPLSAG-----------------L     254
                *:   .*.::.**: :*:*:  .          .:: **.                   *

p50_A.nidu      VGPTPIPAVPTTSAAATSTAVTTPHLEMLSTRVRKLTADAEALASARKRALDSAKAAHSS     339
p50_human       QG-----------------ACLMETVELLQAKVSALDLA----------VLDQVEARLQS     287
                 *                 *     :*:*.::*  *             .**..:*  .*

p50_A.nidu      RHRTASDFSSPIDPEQAAQRDEQATKIQALYATLPTIQSLHPILPSVLERLRSLRAIHAG     399
p50_human       VL---GKVNEIAKHKASVEDADTQSKVHQLYETIQRWSPIASTLPELVQRLVTIKQLHEQ     344
                     .....  . : :.:  :  :*:: ** *:   . :   **.:::** ::: :*

p50_A.nidu      AAQAAESLDELEKRQADMAGEIEQWQEGLKVVEEKMSQSEAALKSNIELVEPWVRDLEKR     459
p50_human       AMQFGQLLTHLDTTQQMIANSLKDNTTLLT-------QVQTTMRENLATVEGNFASIDER     397
                * * .: * .*:. *  :*..:::    *.       * :::::.*:  **  . .:::*

p50_A.nidu      MEKLESGK   467
p50_human       MKKLGK--   403
                *:** .
```

p50-Arp1 in porcine dynactin (PDB 8PTK)

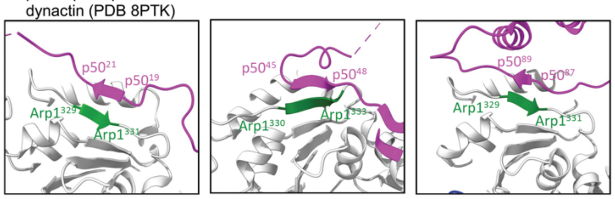

Supplement: Supplement 1 [file NIHPP2026.05.29.728734v1-supplement-1.pdf]
